# Views of Living Donor Liver Transplant Recipients About Xenotransplantation in Türkiye: A Qualitative Study

**DOI:** 10.1111/xen.70139

**Published:** 2026-05-12

**Authors:** Deniz Yavuz Baskiran, Berna Bayır, Halil İbrahim Bilkay, Sezai Yılmaz

**Affiliations:** ^1^ Liver Transplantation Institute Malatya Inonu University Faculty of Medicine Malatya Türkiye; ^2^ Department of Nursing Konya KTO Karatay University School of Health Sciences Konya Türkiye

**Keywords:** liver transplantation, organ transplantation, patient attitudes, qualitative research, xenotransplantation

## Abstract

The aim of this qualitative study is to demonstrate the views of patients who had undergone human‐to‐human liver transplantation towards xenotransplantation. The study included 20 patients who had experienced the transplant waiting process, transplantation surgery, and posttransplant period. The data were collected through in‐depth interviews using a semi‐structured interview form. The findings indicated that patients’ views on xenotransplantation varied between positive, negative, and ambivalent attitudes. While some of the participants indicated that they could accept such transplantation as the last resort with a utilitarian approach that prioritised the maintenance of life, some of them adopted a cautious or negative attitude due to religious beliefs, ethical concerns, and distrust of the unknown. Lack of information came forth as a determinative factor in indecision and dilemma. The views were categorised under the themes of acceptance for sustaining life, religious beliefs and ethical values, seeking trust in the face of uncertainty, psychosocial domain, and perceived opportunities and challenges. Generally, it was concluded that patients’ attitudes towards xenotransplantation were shaped within a conditional acceptance framework and the process should be evaluated multidimensionally.

## 1 Introduction

End‐stage liver failure (ESLF) is a progressive and irreversible clinical picture that leads to significant mortality and morbidity worldwide [1]. Today, liver transplantation represents the only curative treatment modality with proven efficacy for ESLF. A liver can be transplanted from cadaveric donors or living donors. Depending on the sociocultural structures, donation systems, and health policies of the countries, the utilisation rates of these two approaches significantly differ. While cadaveric donation is the predominant system in Western countries, approximately 80–85% of liver transplants appear to rely on living donor liver transplantation (LDLT) in countries with low cadaveric donation rates, such as Korea, India, and Türkiye [2]. According to the Ministry of Health of the Republic of Türkiye 2025 report, more than one million people in the world and approximately 32.000 people in Türkiye have been reported to be awaiting organ transplantation [3]. Although more than 49.000 organs were transplanted in the United States in 2025, the number of patients who were waiting in line for transplantation remained over 100.000, and approximately 13 people lost their lives every day before a suitable organ could be found [4]. These results indicate the inadequacy of allotransplantation for transplantation. The inadequacies and lives lost while waiting for transplant have brought the need for alternative transplant to the agenda. The replacement of human organs with animal organs, that is, xenotransplantation, has been demonstrated as a potential solution to the organ shortage problem. Pigs are considered as the best candidates for xenotransplantation due to their organ physiologies and similarities in size to human organs [5]. However, pigs are considered suitable donors for xenotransplantation not only because of similarities in organ size and physiology with humans but also due to several biological and logistical advantages. These include their short gestation period, large litter size, ease of breeding [6], and the availability of well‐established genetic engineering technologies that allow modification of immunologically relevant genes [5, 6]. Thus, Xenotransplantation using porcine organs has emerged as a promising strategy to help address the persistent shortage of human donor organs. Although significant scientific progress has been achieved in recent years, xenotransplantation remains largely experimental and continues to be investigated as a potential future solution to organ scarcity [7]. Clinical trials involving the transplantation of genetically modified pig kidneys into humans are expected to start in the United States after the food and drug administration (FDA) recently authorized a phaseless trial approach. These xenotransplantation studies represent the result of many years of preclinical work, research conducted in deceased donors, and compassionate‐use procedures carried out in a small number of patients [8]. On the other hand, a study published in 2025 reported that a pig liver was transplanted into a brain‐dead adult in China; the xenograft produced golden coloured bile two hours after portal vein reperfusion and reached 66.5 mL on the tenth postoperative day, and no significant change was reported in immunoglobulin G or immunoglobulin M levels in the preoperative and postoperative period [9]. Despite these advances, it should be noted that xenotransplantation is not only a medical novelty but also a multilayered practice with ethical, religious, cultural, and social dimensions [10]. Given the importance of patient‐centred care, recently emphasised, the necessity to consider the patients’ point of view in the use of pork, deemed religiously and culturally impure by Muslims and Jews [11], in xenotransplantation has appeared [12]. A study evaluating the knowledge and views of Muslim patients about xenotransplantation reported that the patients lacked sufficient knowledge to evaluate this condition from a religious perspective and therefore adopted a negative attitude towards such treatment [13]. Previous studies investigating attitudes toward xenotransplantation have primarily focused on the general public [14], healthcare professionals [15], and student populations [16]. However, an increasing body of literature has begun to explore attitudes among specific patient groups, including individuals with end‐stage kidney disease, transplant candidates, and parents of children with severe cardiac conditions. Recent reviews and empirical studies have highlighted that acceptance of xenotransplantation may vary substantially depending on patients’ clinical experiences, cultural context, and ethical considerations [17, 18]. Nevertheless, qualitative and mixed‐method studies have increasingly explored attitudes among patients and other stakeholders involved in transplantation processes. For example, a focus group study identified various factors influencing perceptions of xenotransplantation clinical trials, such as ethical concerns, perceived risks, cultural beliefs, and trust in medical innovation [19]. A recent qualitative study conducted with organ transplant patients in Türkiye indicated that patients require religious, social, and community support regarding their views on xenotransplantation [20]. Another study conducted in Türkiye indicated that approval for organ transplants from non‐halal animals was quite low among individuals with strong religious beliefs [21]. On the other hand, the number of qualitative studies examining perspectives on xenotransplantation among individuals who have personally undergone the LDLT process remains extremely limited. Recipients of LDLT may represent a particularly informative group in discussions of xenotransplantation because their transplant experience differs from many other transplant populations. Unlike recipients of organs from deceased donors, LDLT recipients often witness the physical and emotional burden placed on a healthy family member who voluntarily undergoes major surgery for donation. This experience may involve complex intra‐family dynamics, feelings of gratitude, indebtedness, and concerns regarding potential donor complications. Such experiences may shape recipients’ ethical reasoning about alternative organ sources and influence how they evaluate emerging options such as xenotransplantation. Therefore, exploring the perspectives of this group may provide unique insights into how individuals who have directly experienced the moral and psychosocial dimensions of organ donation interpret the possibility of animal‐to‐human transplantation. This study aimed to reveal the perceptions, attitudes, and ethical considerations towards xenotransplantation with qualitative methods through semi‐structured interviews with patients who were transplanted with living donor livers at the Liver Transplant Institute of Inonu University (Malatya, Türkiye). It is considered that the study would contribute to the understanding of social acceptance, especially regarding donor safety, religious sensitivities, animal rights, and alternative organ procurement methods.

For this purpose, the following research questions were posed to guide the study:

- How do patients who have undergone LDLT perceive xenotransplantation?
- How do individuals who have undergone liver transplantation from a living donor interpret the concept of xenotransplantation in terms of its level of benefit, from a social perspective, and from a religious perspective?

## 2 Method

### 2.1 Research Design

The study was conducted using a descriptive phenomenological approach grounded in the philosophical foundations of Edmund Husserl, which focuses on understanding individuals’ lived experiences and the meanings they attribute to those experiences [22]. The data obtained from participants’ statements were categorized and analyzed, thereby providing an in‐depth understanding of the thoughts and experiences of individuals. This design was preferred, as it allows the description of the studied case from the participant's perspective and the collection of rich, detailed data [23].

### 2.2 Population and Sample

The study was conducted at the Liver Transplant Institute of Inonu University between October 2025 and January 2026. The population consisted of individuals who underwent human‐to‐human (living/cadaver) liver transplant. To determine the study group, the criterion sampling method—one of the purposeful sampling types—was used. This sampling method involved the cases identified in accordance with the criteria established by the researcher. Accordingly, the study sample consisted of 20 adult patients aged 18 years or older who had undergone a living or cadaveric transplant at İnönü University Liver Transplantation Institute at least one week prior to the study, had not experienced posttransplant rejection, and participants were cognitively able to communicate and provide informed consent. Patients with an additional chronic illness, those undergoing active treatment due to a complication arising after transplantation, or those with a psychiatric problem were excluded from the study. Patients who met the specified criteria were interviewed at random. All suitable patients were seen consecutively. articipants were asked to indicate their level of religious commitment, as it was considered that this could influence their views on xenotransplantation, and all participants identified themselves as Muslim. After being informed about the study, three patients declined to participate. Maximum variation sampling was used to capture diverse perspectives among transplant recipients. Differences in terms of age, gender, donor type (living or deceased) and the time elapsed since transplantation were considered to represent maximum diversity. This approach allowed the study to include participants with differing transplant experiences. Data saturation was considered important. Once maximum diversity had been achieved and the data began to show repetition, it was deemed that saturation had been reached. Data collection continued until thematic saturation was achieved; it was assumed that the data had reached saturation when it became apparent that no new insights were emerging from the interviews and the responses were beginning to show similarities.

### 2.3 Data Collection Process, Validity, and Reliability

In this study, a semi‐structured interview form was used as a data collection tool to determine patients’ personal characteristics and their views on xenotransplantation. The interview form included 4 demographic questions (age, gender, transplant duration, and transplant type) and 8 main semi‐structured questions with 7 follow‐up questions exploring patients’ transplantation experiences, the feasibility and success of xenotransplantation, cultural factors, animal rights, and religious and social considerations. Since no valid and reliable standardized tool suitable for the Turkish population was available, a conceptual framework was developed based on the literature [20, 24] to guide the questions and explore participants’ acceptance, opinions, and suggestions regarding xenotransplantation. The form was finalized after review by two qualitative research experts and a pilot interview with two participants. Interviews lasted 11–23 min, depending on participants’ responses and prior familiarity with the topic. Although shorter than the typical 30–90 min range for qualitative interviews, participants were able to provide rich, meaningful insights. During the interviews, researchers encouraged participants to elaborate on their answers, asked probing follow‐up questions when necessary, and provided clarifications to ensure the depth and richness of the data collected. Additionally, the time spent providing explanations before the interviews began was not included in this duration.

Before the interviews, participants were informed about the purpose of the study, confidentiality principles, and the use of their data solely for scientific purposes. They were told that their responses would be audio‐recorded, anonymized, and coded to protect confidentiality. Interviews were conducted face‐to‐face in a quiet room at the clinic for discharged patients. Participants were first asked about their prior knowledge of xenotransplantation, followed by a brief explanation of its definition, clinical history, type of animal used, and current success rate. Open‐ended questions such as “Do you have anything else you would like to add?” and “What did you think about this matter?” were used to allow participants to express their opinions in detail. Interviews lasted approximately 11–23 min, depending on participants’ responses and prior familiarity with the topic.

To ensure validity and reliability, participants were encouraged to provide clear and unbiased answers, and were informed that there were no right or wrong responses, and their answers would not affect their treatment or be considered evaluations of the transplantation institution or staff. Interviews were conducted only by the researchers to maintain a trust‐based environment, and third parties were not present. All interviews were audio‐recorded and key statements were noted. The researcher who conducted the interview transcribed the data verbatim. Participants were assigned codes to preserve anonymity and avoid bias. During the analysis process, the researchers regularly reviewed transcripts, codes, and themes, and an independent expert also evaluated the resulting themes for appropriateness. Probing questions and clarifications were used throughout to ensure depth and richness of the collected data.

### 2.4 Coding

The interview transcripts were analyzed using thematic analysis. Initial coding was performed independently by two researchers who reviewed the transcripts line‐by‐line to identify meaningful units of data. After the initial coding process, the researchers compared their codes and discussed discrepancies until consensus was reached. In cases where disagreement persisted, a third researcher reviewed the relevant excerpts and contributed to the final coding decision. This collaborative process enhanced the credibility and rigor of the analysis. No formal intercoder reliability metric (e.g., Cohen's kappa) was calculated; however, the collaborative process and consensus discussions ensured consistency and enhanced the credibility and rigor of the analysis.

### 2.5 Data Analysis

MAXQDA 2020 software was used in the analysis of qualitative data. The thematic analysis method defined by Braun and Clarke (2006) was used to classify the participant statements [25]. The data collected from twenty participants were read and transferred to the programme, and then the coding process was initiated. During the coding process, the researchers came together regularly to evaluate the process and short notes were kept to document the decisions made and thoughts. A total of 45 codes were initially created based on the statements of 20 participants, and the document of 20 participants was coded 205 times. After the coding was completed, the researchers reviewed the codes. At this stage, similar and different codes and their compatibility with the research questions were assessed; consequently, 32 codes were merged, and 6 codes were removed. A total of 15 sub‐themes were generated under 5 main themes.

### 2.6 Ethical Considerations

Prior to commencing the study, ethical committee approval (Decision No: 2025/8534, Decision Date: 10/14/2025) and institutional permission were obtained from the Scientific Research and Publication Ethics Committee of Malatya Inonu University. Each stage of the study adhered to the principles of the Declaration of Helsinki. In addition, written and verbal consent was obtained from the participants after they were informed about the study. The participants were informed that their participation in the study was voluntary, their names would be kept confidential, and the data gathered would be used solely for scientific purposes. The names of the participants in the study were coded to protect confidentiality (P1, P2, P3, …, P20).

## 3 Findings

### 3.1 Descriptive Characteristics of the Participants

**FIGURE 1:**
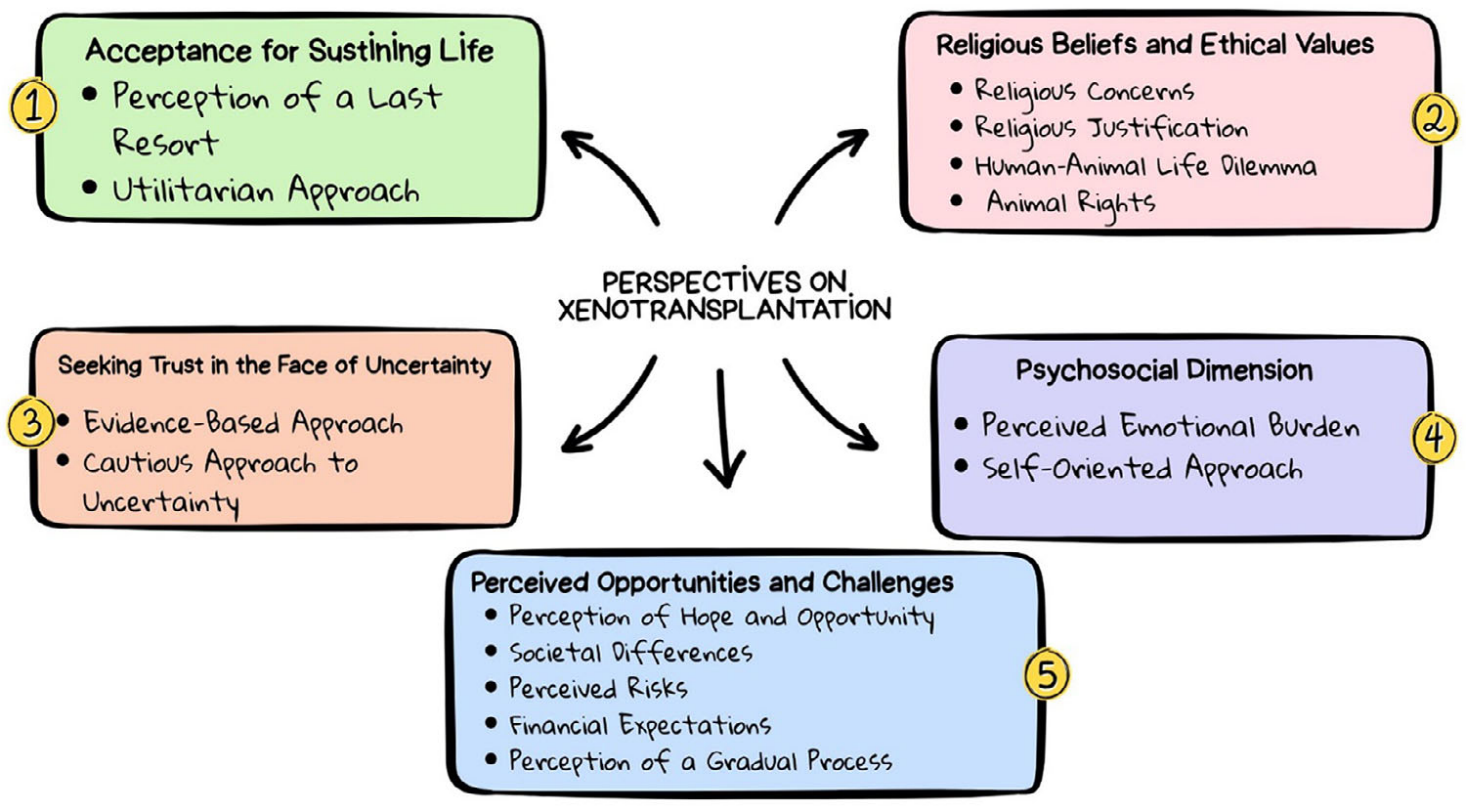
Views of the recipients about xenotransplantation.

The age of participants ranged between 19 and 65 years; three of them were female, and the others were male. When the transplant type was analysed, it was observed that most of the participants received organ transplants from living donors, while fewer participants received transplants from cadaveric donors. Donors in living donor transplants were predominantly first‐ and second‐degree relatives (children, siblings, nephews, and spouses). The time since transplantation ranged from 14 days to 15 years (Table 1). Figure 1 shows the participants’ views about xenotransplantation and the resulting themes.

**TABLE 1:** Descriptive characteristics of the participants.

Participant	Gender	Age	Transplant type	Donor	Transplant duration
P1	Male	65	Living donor	Sibling	2 Years
P2	Male	52	Living donor	Child	2 Years
P3	Male	45	Living donor	Sibling	15 Years
P4	Male	52	Living donor	Child	2 Years
P5	Male	48	Living donor	Child	2 Years
P6	Male	64	Living donor	Child	4 Months
P7	Male	25	Cadaver	−	2 Years
P8	Male	61	Living donor	Child	5 Months
P9	Female	19	Living donor	Sibling	2 Weeks
P10	Male	30	Living donor	Sibling	12 Years
P11	Male	65	Living donor	Nephew	5 Months
P12	Male	55	Living donor	Child	9 Months
P13	Male	47	Living donor	Sibling	3 Years
P14	Male	53	Cadaver	−	6 Months
P15	Male	54	Living donor	Spouse	15 Years
P16	Male	36	Living donor	Nephew	4 Months
P17	Male	62	Living donor	Nephew	12 Years
P18	Female	45	Living donor	Nephew	14 Days
P19	Female	58	Living donor	Spouse	10 Months
P20	Male	30	Cadaver	−	12 Years

I. Acceptance for Sustaining Life

Analysis of participants’ views showed that the procedure was primarily considered for the purpose of sustaining life. Two sub‐themes, perception of a last resort and pragmatic approach, were determined under this main theme.

#### 3.1.1 Perception of a Last Resort

Some of the participants (*n* = 14) described xenotransplantation as an acceptable procedure when all other treatment options have been exhausted. These participants defined the procedure as a final alternative in case of necessity rather than a preferred option.

“I mean, I could have accepted this as the final option. If I had no other option, if I had no chance to get it from a human being, I mean, from a living person or a cadaver, I would accept it.” (P16)

“When things get really hard, people will just have it done. They won't really have a choice — they'll want to stay alive.” (P20)

#### 3.1.2 Utilitarian Approach

- Religious Beliefs and Ethical Values

“If it saves a life in the end, I suppose it's all good.” (P1)

“If this procedure is beneficial to humankind, I will strongly support it.” (P2)

“If anything is beneficial for health and humanity, I strongly support it. I find this study, I mean animal‐to‐human transplantation, quite appropriate.” (P5)

Some of the participants (*n* = 9) evaluated xenotransplantation based on the benefits it would confer. This sub‐theme showed that the lifesaving potential of the procedure at the individual or social level was highlighted.

“If it saves a life in the end, I suppose it's all good.” (P1)

“If this procedure is beneficial to humankind, I will strongly support it.” (P2)

“If anything is beneficial for health and humanity, I strongly support it. I find this study, I mean animal‐to‐human transplantation, quite appropriate.” (P5)

Analysis of participants’ views showed that the procedure was discussed based on religious beliefs and ethical values. Four sub‐themes were identified under this main theme: human‐animal life dilemma, religious concerns, religious justification, and animal rights.

#### 3.1.3 Human‐Animal Life Dilemma

Some of the participants (*n* = 13) evaluated xenotransplantation based on the value of human and animal life. These participants defined human life as more prioritised and sacred than animal life. On the other hand, some participants expressed their discomfort about the harm to animals but indicated that they prioritised their own lives.

“No matter how supreme animal rights are, human rights prevail. Now I prefer to have animals die rather than human beings.” (P15)

“I mean, at the end of the day, that animal is a living being too. It's one of God's creations. But human beings are more sacred.” (P6)

“I believe that God created animals to serve human beings.” (P7)

“It wouldn't sit right with me to see animals get hurt. But at the same time, I haven't really lived my life yet, so I'd put my own life first.” (P2)

#### 3.1.4 Religious Concerns

Some of the participants (*n* = 10) expressed their hesitations about xenotransplantation from a religious point of view. These concerns differed especially depending on the species of animal. Some participants indicated that they strongly rejected transplants from animals that are accepted religiously forbidden, such as pigs. On the other hand, while other participants voiced reservations about animals such as pigs and dogs, they assessed transplants from edible animals to be acceptable.

“The only downside is the religious dimension” (P3)

“Maybe it has a lot of benefits. But people might say no to pigs from a Muslim perspective. I mean, I'm not in favour of pigs… If our religion doesn't allow it, then I wouldn't do it either. Even if I were about to die right now, I wouldn't accept it from a pig.”(P12)

“For example, I definitely wouldn't accept it from a pig. I wouldn't accept it from a dog either. But from another animal whose meat is permissible—like a sheep, a goat, a cow, a lamb… that would be fine. I wouldn't say no to that.”(P17)

“When it comes to pigs, I start to have some doubts. I kind of wonder, you know…” (P19)

#### 3.1.5 Religious Justification

Some of the participants (*n* = 10) used expressions to legitimise xenotransplantation from a religious perspective. These participants indicated that the procedures for health and recovery could be tolerated from a religious point of view.

“I don't believe there would be any religious problems, because we have a hadith that: ‘wherever there is knowledge, go there’.” (P5)

“I believe that most of the medicines we take are not halal, but since they cure people in the world, my Allah will hopefully treat our bodies with them and render them halal.” (P10)

“After all, God has given life to that animal too. If He's putting it in front of me as a cure, then He must also be sparing my life through it. ” (P11)

“For Muslims, pork is haram, but when it is a matter of health, I believe certain things can be tolerated. I mean, there might be a logical explanation. I mean, I believe there is no sin.” (P16)

#### 3.1.6 Animal Rights

- Seeking trust in the face of uncertainty

“If no harm is inflicted on the animal, I mean, if the animal can also live like a human being, then there is no problem. But if that animal dies after taking its organ, it is not okay. It would not be right. It also has a soul and takes breath.” (P12)

“I also think that, just as a human life is precious to us, an animal's life is just as precious to it. The only real difference between animals and humans is that animals can't talk or think the way we do. Otherwise, if they were able to speak, they'd probably say: ‘Don't take my liver. Don't transplant it. And if you're going to take it, then heal me again afterward‘.” (P10)

A few participants (*n* = 3) discussed xenotransplantation from the perspective of animal rights. These participants emphasised that animals should never be harmed and their lives should be protected.

“If no harm is inflicted on the animal, I mean, if the animal can also live like a human being, then there is no problem. But if that animal dies after taking its organ, it is not okay. It would not be right. It also has a soul and takes breath.” (P12)

“I also think that, just as a human life is precious to us, an animal's life is just as precious to it. The only real difference between animals and humans is that animals can't talk or think the way we do. Otherwise, if they were able to speak, they'd probably say: ‘Don't take my liver. Don't transplant it. And if you're going to take it, then heal me again afterward‘.” (P10)

When the participants’ opinions on xenotransplantation were analysed, it appeared that the procedure was perceived as a process that involved uncertainties, and the search for trust stood out accordingly. Two sub‐themes were identified under this main theme: evidence‐based approach and Cautious approach to uncertainty.

#### 3.1.7 Evidence‐Based Approach

Some of the participants (*n* = 8) evaluated the acceptability of xenotransplantation based on scientific evidence and the results of previous procedures. These participants indicated that xenotransplantation would be more acceptable if it had been tested before, if success rates were demonstrated, and if it was socially accepted over time.

“Science and medicine have already researched this; if a disease transmitted from any animal, other than animal transplantation, would cause harm to humans, they already analyse it and transplant it after taking the necessary measures.” (P1)

“I used to be reluctant to try anything I had never heard of before. But if I must undergo transplantation from an animal in the future, and if these transplants have already been performed and have been successful by then, and if 3–5 people have already experienced it and people have become accustomed to it, then maybe I can accept it. I would like to wait and see the success of this transplantation first. I believe it would not be good to speak and do anything unless I see the result.” (P10)

“It should be tried first.” (P18)

#### 3.1.8 Cautious Approach to Uncertainty

- Psychosocial Dimension

“If a disease transmitted from any animal, other than animal transplantation, would cause harm to humans, they already analyse it and transplant it after taking the necessary measures” (P1)

“I suppose that the religious dimension should, of course, be researched.” (P3)

“I would suggest that they should be more careful than human‐to‐human procedures. In other words, I would tell them that they should protect themselves more from infectious diseases and that they should pay attention to their medication and check‐ups.” (P13)

“In the beginning, I would certainly prefer human‐to‐human transplantation because it is a novel process. But for example, if I were told that the organ to be excised from a human being is damaged, but if I prefer, there is also such an option as transplantation from animal to human being, then I would be more likely to prefer that.” (P7)

Some of the participants (*n* = 6) emphasised that preventive and protective measures should be taken against the uncertainties related to xenotransplantation. Accordingly, it was suggested that the medical and religious aspects of the procedure should be thoroughly studied, more caution should be exercised against infectious diseases, and individual follow‐ups should be intensified.

“If a disease transmitted from any animal, other than animal transplantation, would cause harm to humans, they already analyse it and transplant it after taking the necessary measures” (P1)

“I suppose that the religious dimension should, of course, be researched.” (P3)

“I would suggest that they should be more careful than human‐to‐human procedures. In other words, I would tell them that they should protect themselves more from infectious diseases and that they should pay attention to their medication and check‐ups.” (P13)

“In the beginning, I would certainly prefer human‐to‐human transplantation because it is a novel process. But for example, if I were told that the organ to be excised from a human being is damaged, but if I prefer, there is also such an option as transplantation from animal to human being, then I would be more likely to prefer that.” (P7)

When the participants’ views on xenotransplantation and transplant experiences were analysed, it appeared that the process held a significant place for psychosocial effects. Two sub‐themes were identified under this main theme: perception of emotional burden and self‐oriented approach.

#### 3.1.9 Perception of Emotional Burden

Some of the participants (*n* = 8) associated the transplantation process with a heavy emotional burden. This burden was described through feelings of uncertainty, unhappiness, obligation, and gratitude. The participants emphasised the emotional impact of the relationship with the donor, especially in transplants from living donors.

“Now I had to put my child forward. I was transplanted with the organ from my child.” (P6)

“I was transplanted with a donor from a cadaver. I constantly feel a sense of uncertainty. Because there is uncertainty about the surgery, if the transplant is not possible, I will feel strongly unhappy and especially demoralised.” (P7)

“I had no children. If my nephew had not been compassionate, if he were not from a decent family, if he had not donated his liver to me, I would be dead now.” (P11)

“My nephew donated his liver to me. What can I say to him? I owe him for the rest of my life.” (P17)

#### 3.1.10 Self‐Oriented Approach

- Perceived Opportunities and Challenges

“No matter what my spouse or friends think, my health and my life matter more to me. I wouldn't really be in a position to care about what anyone else thinks.” (P4)

“I mean, it is more important to think, ‘I have to get out of this situation; I have to go back to my old life, ’ rather than about what people think. In the end, I believe that a person should think about himself/herself. I mean, if you think, ‘I received it from a human being,’ what can the other person think? I mean, how would they know?” (P7)

Some of the participants (*n* = 3) stated that they prioritised their health and life rather than the reactions from the environment in case of organ transplantation from animals. This sub‐theme showed that individual well‐being and the desire to hold on to life were prioritised over social evaluations.

“No matter what my spouse or friends think, my health and my life matter more to me. I wouldn't really be in a position to care about what anyone else thinks.” (P4)

“I mean, it is more important to think, ‘I have to get out of this situation; I have to go back to my old life, ’ rather than about what people think. In the end, I believe that a person should think about himself/herself. I mean, if you think, ‘I received it from a human being,’ what can the other person think? I mean, how would they know?” (P7)

When the participants’ views about xenotransplantation were analysed, it was observed that the procedure was perceived as a process involving both opportunities and various challenges. Five sub‐themes were identified under this main theme: perception of hope and opportunity, societal differences, perceived risks, financial expectations, and perception of a gradual process.

#### 3.1.11 Perception of Hope and Opportunity

Most of the participants (*n* = 16) considered xenotransplantation as an alternative to shorten the waiting time for organ transplant and to reduce dependence on living donors. The procedure was stated to facilitate the process of finding organs and to provide an alternative for patients who had been on transplant waiting list for a long time.

“It is promising because in such a case, people will not be beholden to anyone. Then… animal organs will be more easily available.” (P4)

“I mean, as far as I know, there are people waiting for their turn for either living donor or cadaveric transplants. There will definitely be an alternative as a solution. It will help.” (P16)

“I guess it's promising for people who have been on transplant waiting list for years.” (P20)

#### 3.1.12 Societal Differences

Some of the participants (*n* = 3) stated that attitudes towards xenotransplantation may differ according to their social and cultural characteristics. Factors such as age and educational level were stated to affect the level of acceptance.

“My neighbours and relatives may think, ‘Oh, do you know that someone has been transplanted from a pig?’ We can hear words such as, ‘You can't drink water from his hand, you can't eat food.’ But as time passes and people hear these things, it may turn into an usual matter.” (P10)

“Intellectual people may accept it, but some people living in rural areas reject it. Younger, more educated people accept it, but those over 40 might cause trouble.” (P12)

#### 3.1.13 Perceived Risks

Some of the participants (*n* = 3) drew attention to the ethical, biological and abuse risks associated with xenotransplantation. These risks included reactions to animal rights, the possibility of commercial or misuse of the procedure, and concerns about tissue compatibility.

“When we consider animal rights, the heads of animal rights may also try to prevent it.” (P4)

“For example, I believe that people might go to the point of slaughtering some animals continuously in order to rejuvenate themselves…. For example, renowned businessmen might turn this into an opportunity and transplant organs every year, I do not know, heart transplants, or that sort of thing.” (P7)

“I don't think it would match human tissue so well.” (P13)

#### 3.1.14 Financial Expectations

Some participants (*n* = 2) emphasised that xenotransplantation should be affordable. It was stated that the procedure could offer an option that neither makes individuals dependent on family members nor on financial resources.

“Even if one depends on their children, this is no way to live, for example. You do not rely on your relatives… money is not involved. So, it would be good.” (P4)

“In fact, I believe the state should spare a budget for this issue. It should support it.” (P5)

#### 3.1.15 Perception of a Gradual Process

One of the participants stated that xenotransplantation requires a multistage and meticulous evaluation process and organs excised from animals should be examined thoroughly and transplanted after various filters.

“It is an animal, after all. They will collect samples from it and analyse it. Who knows how many filters it will pass through? It is necessary to transplant it to a human accordingly….” (P15)

## 4 Discussion

There is a growing recognition that the feasibility of the concept of xenotransplantation has been proven and that this milestone may support the acceptance of a pig liver functioning in a living human being [26]. Following the recent approval of the FDA for non‐phase‐specified clinical trials, it is planned to initiate clinical trials on the transplantation of genetically modified pig kidneys into humans in the USA [8]. However, unlike allogeneic transplantation, xenotransplantation raises unique social and ethical issues. In this sense, both decision makers involved in policy development processes and researchers conducting clinical trials should consider not only the public but also the perspectives of patients, their relatives, and other stakeholders involved in the process [27]. Understanding the nuances that lie behind these perspectives is not merely an academic attempt; it is a prerequisite for the ethical and fair implementation of clinical xenotransplantation. The quantitative data collected so far have been critical in identifying whether there is hesitation, but the survey data provides a limited insight into the complex reasons behind it. Qualitative studies are a critical step to truly understand these perspectives [8]. This study aimed to qualitatively determine the perceptions and thoughts of patients who underwent human‐to‐human liver transplantation regarding xenotransplantation. The data collected from the patients indicated that while both positive and negative opinions towards xenotransplantation were observed, there were also patients who were indecisive or had dilemmas due to a lack of information. The main themes of the opinions were “acceptance for sustaining life, religious beliefs and ethical values; seeking trust in the face of uncertainty; psychosocial dimension; and perceived opportunities and challenges.” Most of the patients appeared to adopt a positive attitude towards this type of transplantation. For the theme of “acceptance for sustaining life,” it was determined that the patients had the opinion that they could accept transplant from a pig as a last resort, or, with a utilitarian approach, they could view this transplantation positively if their lives were at stake. It is possible to find studies in the literature where patients are willing to undergo xenotransplantation as a last resort [19, 20, 28] or if it is successful [8, 29]. However, negative opinions were also found at a low level in the present study. When they were asked the question, “If you were offered the option of animal‐to‐human transplantation, what would be your reaction?” they responded, *“For example, I would never accept it from a pig.”* There were also patients who held negative or sceptical views, such as *“When it comes to pigs, I feel a little bit sceptical there; I wonder if…,” “The only downside is its religious dimension…”* A study conducted in Japan with a healthy sample group reported that when asked how they would feel if they were transplanted from an animal, the majority (77%) of the participants answered that they would feel uncomfortable, and this was attributed to the feeling of discomfort in carrying the organs of an animal in their bodies. It was stated that this approach was mostly associated with the feeling of disgust [27]. Considering the minority of negative opinions in this study, it appears that there is a difference between the studies. This difference is considered to be due to sample differences. In the present study, the participants developed a more utilitarian and pragmatic approach towards organ transplantation from pigs since they were personally ill, were at life risk, went through the emergency process for organ transplantation, and had limited options for other treatment modalities. In this case, individuals stated that they could accept transplantation as a “last resort” despite religious or emotional reservations. On the other hand, the participants in the study conducted with healthy people in Japan approached the issue from a more emotional, cultural, and intuitive perspective (disgust, discomfort), since they were not directly under a life threat. Considering the idea of carrying the organ of an animal in the body as disturbing in this group suggested that moral and emotional reactions were prioritised rather than the risk‐benefit account. On the other hand, a qualitative study conducted by Akboğa and Hobek (2023) with patients on dialysis who were waiting for kidney transplantation in Türkiye reported that the majority of patients could unconditionally accept xenotransplantation [20]. When the findings of the present study were reviewed, the similarity between the studies was reflected in the views that living donor transplantation carried an emotional burden, that it could be legitimate from a religious point of view if it were beneficial, and that it could be a promising practice for survival if it was scientifically proven to be reliable. Statements of the majority of the participants in the same study saying that they could unconditionally accept xenotransplantation [20] indicate that the drive to sustain life is decisive in the approach of patients under life‐threatening conditions about this issue. This finding is compatible with the results of the present study and suggests that patients who bear the physical and psychosocial burden of organ loss and prolonged dialysis may relegate ethical, religious, or emotional reservations to the background. This result suggests that attitudes towards xenotransplantation are shaped by factors such as the severity of the disease, the limitation of treatment options and the need for hope, independent of cultural differences, and that these conditions take precedence. A study conducted by Gordon et al., (2025) with organ transplant patients reported that participants indicated that they would be more likely to take part in a pig kidney trial as the first human trial if the possibility of human kidney transplantation was low and their health condition deteriorated [29]. This result supports the theme of “perception of a last resort” derived from the present study.

At the end of the interviews with the patients, when their perceptions about the harm to animals due to xenotransplantation were assessed, the patients reported a dilemma between human and animal, but in the end, if human life is at stake, the life of animals could be sacrificed. On the other hand, when they considered the issue from a religious point of view, they stated that if it were beneficial for human life, it would be religiously acceptable, with the sub‐theme of “religious justification.” A study conducted by Ozcan et al., (2005) in which healthcare professionals, patients undergoing dialysis and transplant patients were assessed together reported that participants who had just been transplanted and found the procedure religiously acceptable were almost three times more likely to accept xenotransplantation under unfavourable outcomes, and more than eight times more likely to accept it under equivalent outcomes [28]. This corroborates existing Islamic bioethical principles such as “what was forbidden may now be permitted if there is a life‐threatening condition” [10, 30]. A study conducted in the USA revealed a relatively high level of acceptance among Muslim participants when xenotransplantation was offered as a lifesaving treatment. This finding emphasised that medical necessity could override cultural and religious reservations even in minority groups [31]. Besides, the present study also determined that patients might hold negative or sceptical attitudes towards transplantation from pigs with the sub‐theme of “religious concerns”. Likewise, the study by Akboğa and Höbek (2023) also reported that the participants especially expected clear and explicit statements from religious authorities about transplants from animals that were not considered haram. The study emphasised that there was no clear moral and legal agreement on xenotransplantation from an Islamic perspective and the debates persisted on this issue. The authors point out the importance of religious bodies to develop clear ethical‐legal guidelines to resolve these uncertainties; otherwise, individuals may tend to refuse xenotransplantation due to the fear of committing sin [20].

The present study showed that the participants held views about xenotransplantation, which were assessed under the sub‐themes of emotional burden, self‐oriented approach, and social differences, resulting from the effects of social and environmental circles. These views revealed that patients were concerned about the feeling of owing the donor or harming him/her due to transplantation from a living donor, while there was also a self‐oriented approach stating that what others think would not matter if his/her life would be saved. Besides, the sub‐theme of ‘societal difference’ indicated that there was a perception that the recipient would be socially marginalised due to transplantation from a pig and that problems such as stigma with the perception of disgust could be experienced. Likewise, in their study Akboğa and Hobek (2023) reported that patients were concerned about exclusion by society even though they expressed that they did not care whether society would support them or not [20]. On the other hand, another study conducted with parents of children with congenital heart failure reported that xenotransplantation was considered favourably as a last resort, but they were concerned that their children would be exposed to stigma for this reason [19]. Organ transplants between bodies of different racial groups raise debates that require reconsideration of both the biological foundations and cultural dimensions of the concept of race [32].

## 5 Conclusion

The present study tries to understand the thoughts of individuals who went through the liver transplantation process in all its stages towards xenotransplantation, making visible not only their opinions on a medical innovation but also a multilayered world of perception shaped on the axis of life, death, hope, and values. The findings suggest that xenotransplantation is not considered by patients as a one‐dimensional medical option but as an existential and social ethical threshold. A significant part of the participants expressed that they could accept xenotransplantation as a potential option with a pragmatic perspective that prioritises the sustainability of life. In particular, the uncertainty caused by the lack of human organ availability and the attrition of the waiting process have emerged as one of the main determinants of this approach. This suggests that the concept of ‘last resort’ may play an influential role in the decision‐making processes of patients in this study. For some participants, the desire to sustain life provided a framework that allowed for the reinterpretation of religious or ethical boundaries. However, positive attitudes towards xenotransplantation were not absolute but rather appeared to be intertwined with serious hesitations and dilemmas. Religious beliefs, ethical concerns, the thought of violating animal‐human boundaries and uncertainties about long‐term outcomes raised strong question marks in the minds of patients. In particular, the lack of sufficient information drove the participants beyond a definite acceptance or rejection into a waiting zone characterised by indecision. This finding suggests that the lack of information creates not only a cognitive burden but also an emotional and moral burden. The psychosocial dimension emerged as another determinative factor in the perception of xenotransplantation. The patients’ self‐perception, their concerns about how they would be perceived by society and the idea of being ‘different’ deepened their emotional reactions towards this type of transplantation. Although the patients tend to adopt a positive attitude in general, it appears that this positive attitude is conditional, questioning and fragile. The findings indicate that providing information, ethical counselling, and psychosocial support could be important considerations for patients in the context of xenotransplantation. Accordingly, it may be beneficial to consider not only biomedical safety but also patients’ information needs, religious and ethical sensitivities, and psychosocial adaptation processes when planning the potential clinical implementation of xenotransplantation. The development of clear, understandable, and unbiased information programmes for transplant candidates would be an important step in lowering their indecision and anxiety levels. Furthermore, a comparative analysis of the opinions of patient groups from different cultural and religious backgrounds in future studies may contribute to the formulation of more inclusive policies to improve the social acceptance of xenotransplantation.

## 6 Limitations

The findings of this study should be evaluated within some limitations. First, the qualitative research design of the study and the limited number of patients who underwent liver transplantation in the sample limit the generalisability of the results. The majority of participants were male, which may limit the transferability of the findings to female transplant recipients. Additionally, detailed information on the underlying etiology of liver failure (e.g., viral hepatitis, autoimmune disease, acute liver failure, or toxic injury) was not consistently available for all participants, which may limit the interpretation of findings, as clinical course and etiology could influence patients’ attitudes toward xenotransplantation. The selection of participants from a single centre may not have reflected the perspectives of patients treated in different health institutions and in different sociocultural contexts. Furthermore, since xenotransplantation has not yet been widely experienced in clinical practice, the views of the participants were largely based on assumptions, personal beliefs, and limited knowledge. This raises the possibility that the opinions expressed may not fully represent attitudes during a real decision‐making process. Furthermore, the tendency to respond in a socially acceptable manner during the interviews may have prevented some participants from fully expressing their genuine opinions, especially when referring to religious and ethical issues. Finally, the collection of data over a certain period ignores the fact of changes in patients’ opinions over time, in accordance with new scientific advancements and social debates. Therefore, the findings of the study should be considered as an evaluation that represents patient perceptions of xenotransplantation within a specific context and time frame. Another limitation of the study is the relatively short duration of the interviews (11–23 minutes). The focused nature of the interview questions and the clinical condition of some participants shortly after transplantation may have influenced the length of the interviews. Interviews were conducted at the transplant center, which could potentially introduce perceived authority bias. However, a trust‐based environment was established, and participants were informed that there were no right or wrong answers, which may have helped mitigate this effect.

## Author Contributions

**Dr. Deniz Yavuz Başkıran**: The researcher who has made significant contributions to the concept or design of the study; to the acquisition of data for the study; and to the final approval of the version to be published; and who is responsible for all aspects of the study to ensure that questions regarding the accuracy or completeness of any part of the study are properly investigated and resolved. **Dr. Berna Bayır**: The researcher contributed to the preparation of the draft article, the review of the literature, the design and writing of the methods section, the preparation of audio recordings for analysis, and the critical review of significant intellectual content. **Dr. Halil İbrahim Bilkay**: The researcher has been involved in the analysis of the data obtained and the writing of the findings section of the manuscript, as well as in methodological arrangements. **Prof. Sezai Yılmaz**: The researcher made significant contributions to the concept or design of the study; to the acquisition of data for the study; and to the final approval of the version to be published.

## Funding

The authors have nothing to report.

## Disclosure

This research has not been sent to any other journal. It has not been published anywhere before.

## Ethical Considerations Statement

This study was conducted in full compliance with the principles of research and publication ethics. All data were collected in accordance with ethical standards, with due regard to confidentiality and voluntary participation. Prior to commencing the research, ethical committee approval (Decision No: 2025/8534, Decision Date: 14/10/2025) and institutional permission were obtained from the Scientific Research and Publication Ethics Committee of Malatya İnönü University. Informed consent was obtained from all participants, and strict attention was paid to the protection of personal data and confidentiality. No scientific misconduct such as plagiarism, fabrication, falsification, duplication, salami slicing, or unfair authorship was involved in this study. All sources used have been properly cited in accordance with academic standards. With this statement, I hereby confirm that all stages of the study were carried out in accordance with scientific ethical principles and academic integrity.

## Consent

Consent was obtained from the participants to participate in the study.

## Conflicts of Interest

The authors declare no conflicts of interest.

## Supporting information




Supporting Information

## Data Availability

The data that support the findings of this study are available on request from the corresponding author. The data are not publicly available due to privacy or ethical restrictions.
