# Supplementary material for: Views of Living Donor Liver Transplant Recipients About Xenotransplantation in Türkiye: A Qualitative Study
Source: Xenotransplantation. 2026 May 12;33:e70139. doi: 10.1111/xen.70139 (PMC13162297; doi:10.1111/xen.70139)
Supplement: Supplementary file 1 — Supporting Information [file XEN-33-e70139-s001.docx]

**Patient Consent**This study is being conducted to explore the thoughts of patients who have undergone liver transplantation regarding xenotransplantation. Data will be collected through notes taken during individual interviews with participants who agree to take part in the study. The ultimate goal is to report the analysis results and contribute to scientific publications.

By agreeing to participate, you are consenting to take part in the interviews involved in this process. Participation in this study is entirely voluntary, and you have the right to withdraw at any point during the research. This study is only interested in your opinions. Your answers will not be evaluated as right or wrong. You are free to ask questions or respond openly to the interview questions as you wish.

**Do you consent to participate in this study?**
a) Yes (Consent obtained via audio recording)

**Patient Information Form**

1. How old are you? ...................
2. What is your gender? ...................
3. When did you undergo organ transplantation? ...................
4. What type of transplant did you receive? ...................

**Semi-Structured Interview Form**
Xenotransplantation is the process of transplanting cells, tissues, or organs from one species to another. The area most frequently studied in medicine is the transplantation of pig organs into humans, as pig organs most closely resemble human organs in terms of size and function. The main goal is to address the shortage of donors for patients awaiting organ transplants.

1. Could you tell me a little about your own experience with organ transplantation?
2. Have you heard about organ transplantation from animals to humans before?

a) Yes b) No

1. If your answer is yes, could you briefly summarize what you know about it?
2. If the option of an animal-to-human transplant were offered to you, would you accept it?

a) Yes ……………… Please explain. b) No…………… Please explain.

1. What are your positive views regarding animal-to-human tissue or organ transplantation?

9.1. What do you think could be the advantages of this procedure? In other words, how could this procedure benefit patients?

1. What are your negative thoughts regarding animal-to-human tissue or organ transplantation?

10.1. In your opinion, are there any disadvantages to this procedure? Are there any concerns or ideas you have heard or thought about regarding this?

10.2. Do you think there is any religious objection to this? (At this point, remind the participant that the transplant would involve a pig.)

10.3. From a religious perspective, how do you think your neighbors or relatives would react to this?

10.4. Considering animal rights, what are your thoughts on this type of transplantation?

1. If the option of an animal-to-human transplant had been presented to you before your transplant, what would you have chosen?

11.1. And why do you think that way?

1. In your opinion, is this new method a promising approach for patients?

12.1. And why do you think that? Please explain.

1. If you met someone who was going to undergo an animal-to-human transplant, what advice would you give them?

Thank you for your participation. Finally, if there is anything you would like to say, please feel free to share.
